# Oxidative Stress and Inflammation in Retinal Degeneration

**DOI:** 10.3390/antiox10050790

**Published:** 2021-05-17

**Authors:** Ravirajsinh N. Jadeja, Pamela M. Martin

**Affiliations:** 1 Department of Biochemistry and Molecular Biology, Medical College of Georgia, Augusta University, Augusta, GA 30912, USA; rjadeja@augusta.edu; 2 Department of Ophthalmology, Medical College of Georgia, Augusta University, Augusta, GA 30912, USA; 3 James and Jean Culver Vision Discovery Institute, Medical College of Georgia, Augusta University, Augusta, GA 30912, USA

Inflammation and oxidative stress play prominent roles in the pathogenesis of many degenerative diseases of the retina, such as age-related macular degeneration (AMD), diabetic retinopathy (DR), retinal vein occlusion, and retinitis pigmentosa [1,2,3]. Healthy retinal cells are continuously exposed to high levels of oxidative stress as a normal consequence of significant light exposure and visual signal transduction pathways that generate considerable amounts of reactive oxygen species [4]. However, in aging and/or disease the efficiency of the normal homeostatic mechanisms that exist to counter the potentially deleterious effects of this stress often decline. This disrupts the balance between pro- and anti-oxidative signaling and leads to excessive oxidative stress, related inflammation, dysregulated immune responses, potential blood–retinal barrier compromise, and tissue damage [1,2,4]. Thus, understanding better the mechanisms governing the cellular and molecular events that underlie the switch that precipitates the failure of the retina to respond adequately to oxidative and/or inflammatory insults may support the discovery of new therapeutic targets to prevent and treat irreversible vision loss and blindness. This special issue is a collection of eight original research articles and one review article focused on various aspects of oxidative stress and inflammation in the pathogenesis of retinal degeneration, identification and exploration of novel targets, and development and testing of antioxidant and anti-inflammatory therapies.

The retinal pigment epithelium (RPE), a highly specialized, polarized epithelial cell layer, is situated such that its apical side closely approximates the outer segments of the photoreceptors while its basal side juxtaposes Bruch’s membrane [5]. This sandwiched arrangement facilitates the unique and diverse functions of RPE that are in turn, pivotal for maintaining normal vision, and in particular, central visual acuity [6]. RPE impairment significantly contributes to age-related macular degeneration (AMD) [6]. Further, oxidative stress and inflammation are thought to play major causative roles [7]. Two studies in this Special Issue focus on the detrimental role of oxidative stress in RPE health and retinal degeneration. RPE cells are chronically exposed to a pro-oxidant microenvironment throughout their life. Macchioni and colleagues [8] created an in vitro experimental condition in which human RPE cells (ARPE-19) were exposed to 10 μM H_2_O_2_ (hydrogen peroxide) for several passages to mimic chronic in vivo oxidative stress conditions. It was observed that this type of long-term oxidative insult induced senescence in RPE cells without affecting cell proliferation. Global proteomic analysis revealed a dysregulated expression in proteins involved in the antioxidant response, mitochondrial homeostasis, and extracellular matrix organization. Interestingly, in response to additional pro-inflammatory insults, senescent RPE cells underwent an exaggerated inflammatory reaction. These results indicate senescence as an essential link between chronic oxidative insult and detrimental chronic inflammation. Also with the intent of understanding mechanisms governing the response of RPE to pro-oxidant insult, Martinez-Gil et al. [9] used a variety of methods including proteome array, ELISA, qPCR, and Western blot to evaluate the role of CYP2E1 (Cytochrome P450 2E1) in ethanol (EtOH)-induced oxidative stress in RPE cells. These authors found that EtOH-induced oxidative stress modifies biomarkers of inflammation and angiogenesis. Specifically, ethanol at 600 mM concentration significantly increased ROS levels and upregulated the CYP2E1 expression, thus, promoting cell death. Further, EtOH increased matrix metalloproteinases levels and angiogenic regulators. Subsequently, treatments with N-acetylcysteine (NAC) and diallyl sulfide (DAS) reduced oxidative stress and improved cell survival by modulating the upstream angiogenesis and inflammatory regulators. Overall, this study provided important information—that CYP2E1 upregulation could aggravate retinal degeneration, and that antioxidants could be used as an adjuvant therapy to mitigate it.

Given the abundance of clinical and experimental evidence pointing to oxidative stress as a major player in RPE damage and outer retinal dysfunction, therapeutic interventions that reduce oxidative stress in RPE cells represent a viable option to mitigate retinal degeneration. Three research articles from this special issue, including our own, evaluated the efficacy of different dietary, nutraceutical, and/or pharmacological compounds in limiting oxidative stress in RPE. We evaluated the effects of selenomethionine (Se-Met), the main form of selenium in the diet, on system xc- expression and functional activity and cellular levels of glutathione in cultured RPE cells [10]. We observed that Se-Met activated Nrf2 (nuclear factor erythroid-2-related factor 2) and induced the expression and function of xc- in RPE, providing a robust antioxidant response. Further, the effect of Se-Met on xc- was associated with an increase in maximal velocity and in substrate affinity. Interestingly, Se-Met increased the cellular levels of glutathione in the control, an oxidatively stressed RPE. Overall, this study demonstrated that Se-Met enhances the antioxidant capacity of RPE by inducing the transporter xc- with a consequent increase in glutathione. Hence, dietary Se-Met supplementation could be a viable therapeutic strategy for retinal degenerative diseases. Clementi et al. investigated the protective effect of punicalagin (PUN), the major ellagitannin in pomegranate, on mitochondrial dysfunction associated with H_2_O_2_-induced oxidative stress [11]. Human RPE cells (ARPE-19) were exposed to H_2_O_2_ alone or in combination with PUN to evaluate the effects on cell viability, mitochondrial reactive oxygen species (ROS) levels, mitochondrial membrane potential, respiratory chain complexes, and caspase–3 enzymatic activity. Their results demonstrated that PUN supplementation significantly improved cell viability, maintained a healthy mitochondrial membrane potential, and reduced ROS production. The authors concluded that PUN might be considered a useful nutraceutical agent in treating oxidative-stress-induced RPE degeneration. Chan and colleagues compared the effects of metformin and AMPK (AMP-activated protein kinase) activator, A769662, on sodium iodate (NaIO_3_)-induced oxidative stress and cell death [12]. These authors observed that A769662 provided superior protection against NaIO_3_-induced cytotoxicity compared to metformin. Neither of the drugs affected mitochondrial ROS production or membrane potential. However, interestingly, NaIO_3_-induced mitochondrial fission and inhibition of mitochondrial respiration were reversed by A769662 but not by metformin. In conclusion, it was reported that AMPK activation could exert cytoprotection by restoring mitochondrial respiration and reducing mitochondrial fission.

The age-dependent accumulation of lipofuscin in the RPE is associated with the development of AMD [13]. A significant component of lipofuscin is the bis-retinoid N-retinylidene-N-retinylethanolamine (A2E). Mitochondrial DNA (mtDNA) damage has been identified as an important contributing factor in retinal-degeneration-related pathologies [14]. Continuous mitochondria stress can alter their genome leading to retinal degenerations. The major goal of Donata et al.’s study was to identify mtDNA variants induced by N-retinylidene-N-retinylethanolamine (A2E) exposure along with the molecular mechanisms responsible for retinal degeneration [15]. A variant analysis comparison of transcriptome profiles was evaluated in RPE cells treated with A2E and in untreated cells. An increased number of variants were observed following the A2E treatment. Interestingly, variants mainly occurred within mtDNA coding sequences. Further analysis revealed the involvement of all oxidative phosphorylation complexes, suggesting compromised ATP production. Based on the above, the authors proposed that their observations could be incorporated into clinical diagnostic settings to drastically improve precision molecular medicine in retinal degenerative diseases.

Recently emerging shreds of evidence have highlighted an association between AMD and periodontal disease (PD); however, the underlying mechanisms are poorly understood [16]. Therefore, Arjunan et. al. set out to develop a simple, reproducible model that emulates characteristics of both AMD and PD [17]. The authors evaluated the potential role of oral infection (ligature-enhanced) with the keystone periodontal pathogen *Porphyromonas gingivalis* in the progression of laser-induced choroidal neovascularization (Li-CNV) in a mouse retina. The authors observed inflammatory drusen-like lesions, reduced retinal thickness, and increased vascular leakage in AMD+PD mice retinas using histological and various functional analyses. Further, these pathological changes were associated with significant increases in oxidative stress, angiogenesis, and pro-inflammatory mediators. Collectively, this is the first in vivo study demonstrating a significant role of periodontal infection in the augmentation of AMD phenotype.

Lastly, this special issue contains one research article and a review article focused on diabetic retinopathy (DR). In their review, Yumnamcha et. al. focused on current literature on the contribution of the dysregulation of glycolysis to DR [18]. In the retina, glucose homeostasis is fine-tuned by the tightly regulated interplay between glycolysis, the Krebs cycle, and oxidative phosphorylation that maintains the various metabolic intermediates needed for the various physiological function of the retina. However, hyperglycemia disturbs the different metabolic pathways, disrupting the metabolism and functioning of multiple cell types in the neuro-vascular retina, eventually leading to DR. Future studies using metabolomics approach could pave the way to the development of future therapeutic targets for the prevention and treatment of DR. Along similar lines, Ravera et al.’s in vitro study evaluated the effects of two antidiabetic drugs, metformin (Met) and glibenclamide (Glb), on outer rod segment ectopic aerobic metabolism [19]. Exposure of rod outer segments (OS) to light results in the production of free radicals via aerobic metabolism. It was observed that metformin enhanced complex I activity and ATP production at low concentrations (15 and 150 μM) while reducing the same at higher concentrations (1.5, 2, or 5 mM) in bovine OS cultures. Conversely, treatment with Glb negatively affected the activity of both complexes I and III, resulting in reduced ATP production. Further, the combination treatment significantly reduced aerobic metabolism and oxidative stress production. Collectively, the authors concluded that Met and Glb alone or in combination could be used to modulate energy metabolism in early to advanced DR.
